# Crystal growth of clathrate hydrate formed with H_2_ + CO_2_ mixed gas and tetrahydropyran

**DOI:** 10.1038/s41598-021-90802-6

**Published:** 2021-05-31

**Authors:** Meku Maruyama, Riku Matsuura, Ryo Ohmura

**Affiliations:** Department of Mechanical Engineering, Keio University, 3-14-1 Hiyoshi, Kohoku-ku, Yokohama, Kanagawa 223-8522 Japan

**Keywords:** Physical chemistry, Chemical engineering

## Abstract

Hydrate-based gas separation technology is applicable to the CO_2_ capture and storage from synthesis gas mixture generated through gasification of fuel sources including biomass. This paper reports visual observations of crystal growth dynamics and crystal morphology of hydrate formed in the H_2_ + CO_2_ + tetrahydropyran (THP) + water system with a target for developing the hydrate-based CO_2_ separation process design. Experiments were conducted at a temperature range of 279.5–284.9 K under the pressure of 4.9–5.3 MPa. To simulate the synthesis gas, gas composition in the gas phase was maintained around H_2_:CO_2_ = 0.6:0.4 in mole fraction. Hydrate crystals were formed and extended along the THP/water interface. After the complete coverage of the interface to shape a polycrystalline shell, hydrate crystals continued to grow further into the bulk of liquid water. The individual crystals were identified as hexagonal, tetragonal and other polygonal-shaped formations. The crystal growth rate and the crystal size varied depending on thermodynamic conditions. Implications from the obtained results for the arrangement of operating conditions at the hydrate formation-, transportation-, and dissociation processes are discussed.

## Introduction

The elevation of CO_2_ emissions in the atmosphere is a central, common concern worldwide both in environmental and political manners. Various industrial processes discharge CO_2_-including synthesis gas, which entails corresponding CO_2_ capture and storage (CCS) technique. One approach is “pre-combustion” process, through which CO_2_ is captured prior to the fuel combustion in power plants. This pre-combustion capture can be applied to separate CO_2_ from syngas mixture generated through gasification of fuel sources (e.g., coal, natural gas, and biomass). Particularly, the development of biomass energy may promote the reduction of greenhouse gas emissions^1–3^. Through biomass gasification processes, syngas mixture of H_2_, CO_2_, CH_4_, CO is produced^4,5^. After water–gas-shift reaction, this syngas mixture can subsequently be converted into H_2_ + CO_2_ gas mixture^6^, which requires CCS process. For this purpose, an emerging method is under development, a process utilizing clathrate hydrate crystallization^7^.

Clathrate hydrates (hydrates) are crystalline inclusion solids of cage-like hydrogen-bonded host water molecules encaging guest molecules of gaseous or organic compounds, which form under definite thermodynamic conditions. Hydrates have several unique properties including large capacity of gas storage^8–10^ and guest compound selectivity^11,12^, both of which render an undertaking of hydrate-based gas separation feasible. In addition, any hydrate-based industrial application is harmless to the environment, for the main constituent of hydrate is water molecules, which produces no toxic chemicals during the formation, transportation and decomposition process.

As an alternative CCS method to two major existing technologies, namely chemical absorption^13,14^, which can be toxic and harmful to the ecosystem, and membrane separation^15,16^ necessitating frequent maintenance and accordingly high cost, hydrate-based gas separation is one burgeoning technology. Moreover, CO_2_ capturing by hydrate-based gas separation has comparable to or even lower energy consumption than chemical absorption or membrane separation^17^. Previous studies have shown a great amount of potential of this line; various experiments have been conducted using batch, semi-batch type and continuous separation methods^17–20^. Among these three methods of separation, continuous separation is best suited for industrial use^20^; while batch-type operations involve several sets of reactors to produce purified gas continuously, in the continuous operation, a series of procedures from hydrate formation via discharge of hydrate slurry to hydrate dissociation suffice a single set of reactors and all these procedures are performed concurrently. Therefore, the capital investment for plant construction and operating costs are to be lessened when introducing the continuous separation system. Horii and Ohmura^20^ performed continuous CO_2_-separation experiments in the H_2_ + CO_2_ + water and H_2_ + CO_2_ + tetra-*n*-butylammonium bromide (TBAB) + water systems. Kiyokawa et al.^17^ reported an improved CO_2_-separation performance in the system of H_2_ + CO_2_ + tetrahydropyran (THP) + water. THP is one of the large-molecule guest compounds (LMGC) with water solubility to the hydrate forming system, working as a thermodynamic promoter to moderate the equilibrium pressure. Table 1 summarizes mole fractions of H_2_ and CO_2_ after repetitions of operation for 38 h in the gas phase and in the hydrate-slurry phase from these experimental studies. As these results demonstrate, the separation process adding THP in the system matches a practical operation most advantageously of the three systems, because of the fact that the CO_2_ mole fraction in the gas phase reaches the lowest value, that is, separation efficiency of this system is the highest, and that the H_2_ mole fraction in the slurry phase falls to 0.00, to rephrase it, purified CO_2_ can be obtained through the capture and storage process.

**Table 1:** Summary of experimental results of continuous CO_2_-separation studies.

Authors	System	Feed gas composition by mole fraction	Experimental condition		Results		Ref
			Temperature (K)	Pressure (MPa)	H_2_:CO_2_ mole fraction in the gas phase	H_2_:CO_2_ mole fraction in the slurry phase	
Horii and Ohmura	H_2_ + CO_2_ + water	H_2_:CO_2_ = 0.6:0.4	271.8	7.0	0.87:0.13	0.00:1.00	^20^
Horii and Ohmura	H_2_ + CO_2_ + TBAB + water	H_2_:CO_2_ = 0.6:0.4	277.4	5.0	0.81:0.19	0.18:0.82	^20^
Kiyokawa et al	H_2_ + CO_2_ + THP + water	H_2_:CO_2_ = 0.6:0.4	277.7	5.0	0.92:0.08	0.00:1.00	^17^

For the design of the hydrate formation-, transportation- and dissociation processes to realize, hydrate crystal growth dynamics and crystal morphology need to be clarified. As previous studies have shown, hydrates are usually formed at the interface or in the bulk of the water phase^21–25^. Therefore, one method to multiply the amount of hydrate formation is the expansion of the interfacial area. Crystal morphology, which indicates crystal shape and size, affects dehydration and transportation energy efficiencies of hydrate slurry; when hydrate crystals are small in size, the pressure loss during the transportation of hydrate slurry increases and the dehydration efficiency of the slurry decreases^26^. In the dissociation process, hydrate crystals with large surface areas per unit volume such as dendritic crystals decompose promptly owing to the increase of the overall rate of heat transfer, whilst hydrate crystals with small surface areas such as polygonal crystals remain undissociated for a longer period of time. Thus, understanding of crystal growth dynamics and crystal morphology forms the foundation of hydrate-based gas separation technology.

Previous experimental studies have revealed that crystal growth dynamics and crystal morphology of hydrate are resultant from the characteristics of the guest compounds and thermodynamic conditions of the hydrate-forming system. Ohmura et al.^27^ performed crystal growth observations for CO_2_ hydrate that covers the CO_2_-water interface and forms a film. They demonstrated that the crystal morphology ranges from dendritic to skeletal or polyhedral depending on thermodynamic conditions. Ueno et al.^28^ observed CH_4_ + CO_2_ hydrate crystal growth at the gas/liquid interface and in liquid water. They suggested that the crystal morphology is determined by the guest composition in the liquid phase. Akiba et al.^29^ performed crystal growth observations on the surface of a liquid droplet of TBAB aqueous solution exposed to CO_2_ gas. Ozawa and Ohmura^30^ observed the formation and growth of methane + THP hydrate crystals, clarifying that the morphology varies from pyramidal to an “oligo-polygon” depending on thermodynamic conditions. However, there is no report of crystal growth in a system with H_2_ + CO_2_ gas in preceding studies.

In the present work, visual observations of crystal growth of hydrate formed with H_2_ + CO_2_ mixed gas, liquid THP and liquid water were conducted under different thermodynamic conditions. This study draws a bead on contributing to the industrial utilization of hydrate-based H_2_/CO_2_ separation with a crystallographic approach.

## Results and discussion

### Crystal growth dynamics

We conducted several experimental runs under each experimental condition for the confirmation of reproducibility and demonstrate the representative results here. THP and water are partially soluble. Therefore, partial solubility between THP and water leads to phase separation (liquid THP-liquid water). The mutual solubility data of THP and water, reported by Stephenson^31^, are demonstrated in Table S1. The mole fraction of THP in the aqueous phase is calculated to be 1 − 0.112 = 0.888. The mole fraction of water in the aqueous phase is calculated to be 1 − 0.023 = 0.977. The measured equilibrium conditions, which were obtained experimentally in the present study, and the corresponding gas compositions in each experimental system are summarized in Table 2. We defined the subcooling temperature Δ*T*_sub_ as a difference between the phase equilibrium temperature of the hydrate-forming system and the experimental temperature (Δ*T*_sub_ ≡ *T*_eq_ − *T*_ex_), which is an index of driving force for the crystal growth. Crystal growth observations were conducted under several Δ*T*_sub_ conditions, namely, from Δ*T*_sub_ = 1.3 K to Δ*T*_sub_ = 7.1 K. We determined the experimental conditions as to prevent the formation of H_2_ + CO_2_ binary hydrate.

**Table 2:** Equilibrium conditions and gas compositions in each experimental system.

Figure number	Equilibrium condition		H_2_:CO_2_ gas composition (mole fraction)
	*T*_eq_ (K)	*P* (MPa)	
1, 2	285.5	5.00	0.55:0.45
3	286.6	5.00	0.55:0.45
4	287.1	5.42	0.55:0.45
5	286.2	5.08	0.65:0.35

**Figure 1:**
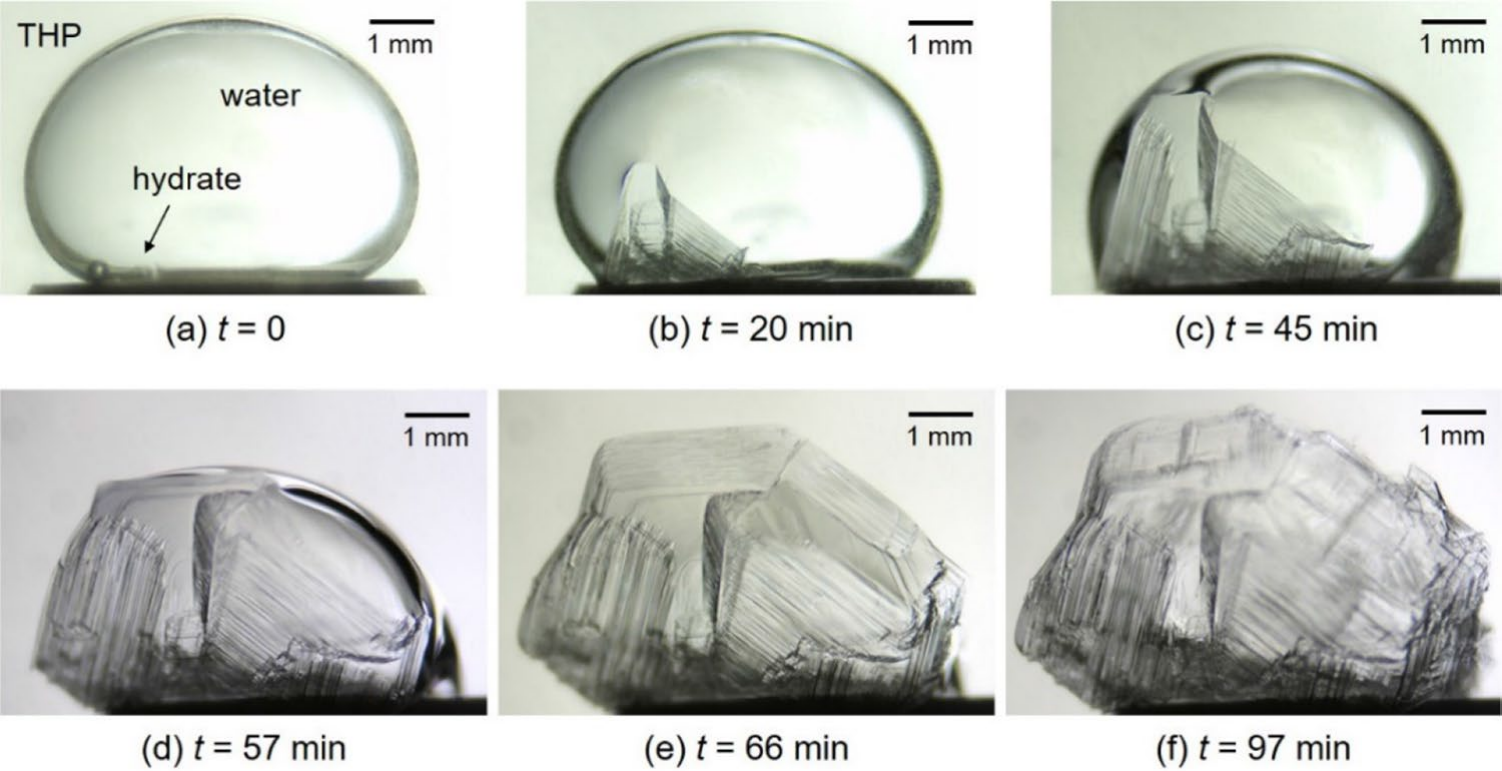
Sequential images of H_2_ + CO_2_ + THP hydrate growing on a water droplet at *T*_ex_ = 284.2 K, Δ*T*_sub_ = 1.3 K, *P* = 5.00 MPa. The gas composition was H_2_:CO_2_ = 0.55:0.45 in mole fraction. The elapsed time after the hydrate nucleation is indicated below each image.

**Figure 2:**
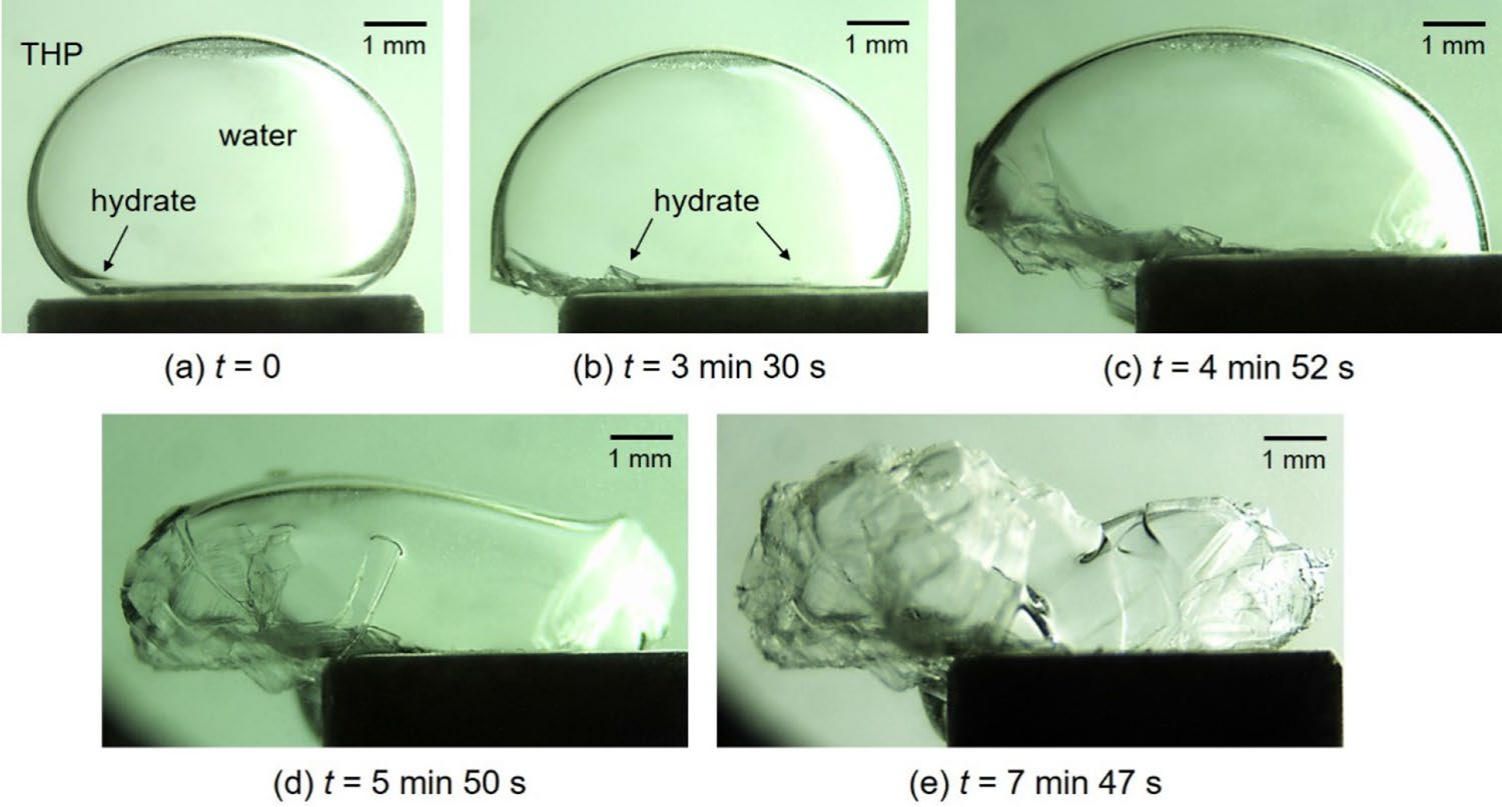
Sequential images of H_2_ + CO_2_ + THP hydrate growing on a water droplet at *T*_ex_ = 283.0 K, Δ*T*_sub_ = 2.5 K, *P* = 5.00 MPa. The gas composition was H_2_:CO_2_ = 0.55:0.45 in mole fraction. The elapsed time after the hydrate nucleation is indicated below each image.

**Figure 3:**
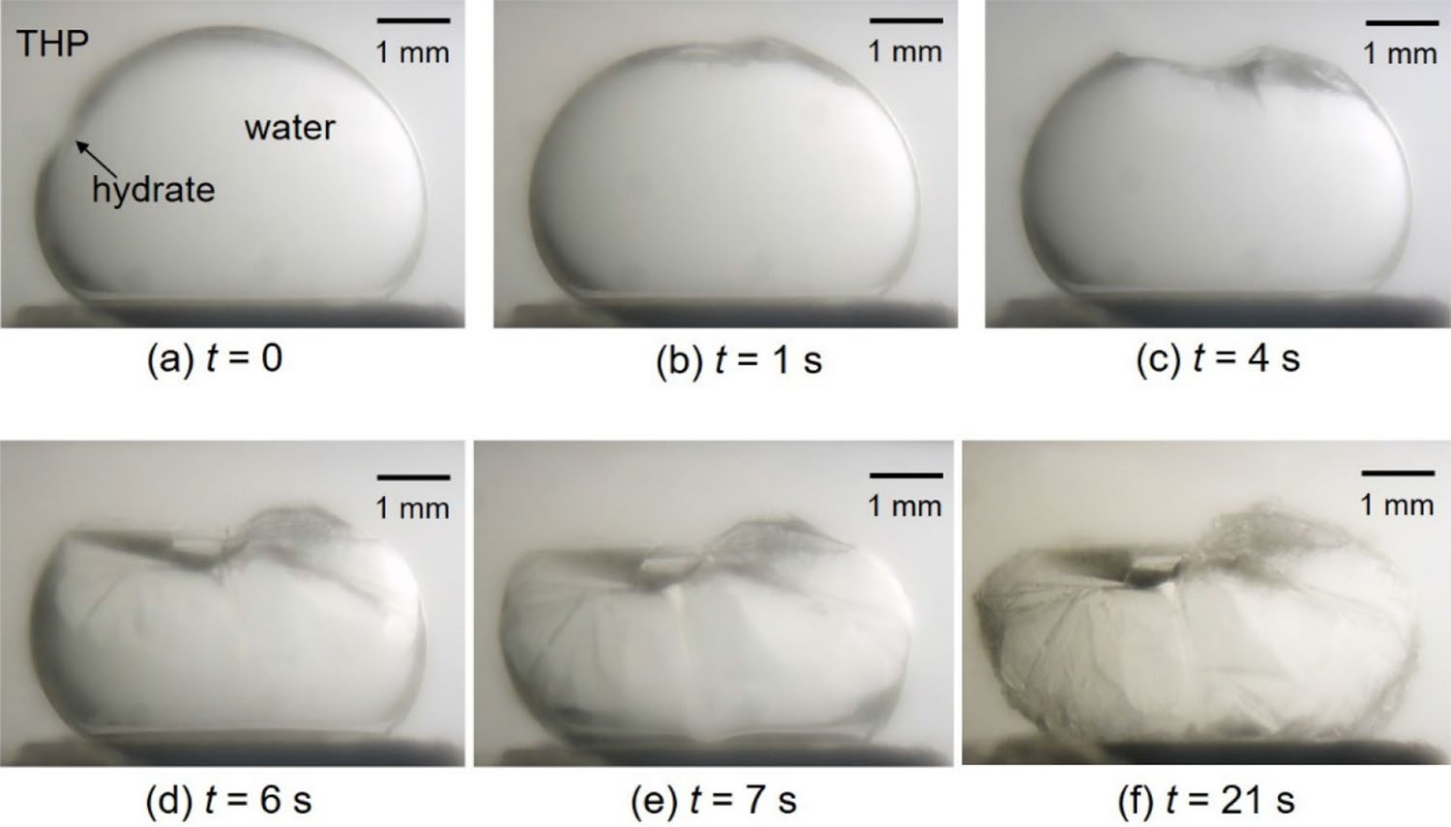
Sequential images of H_2_ + CO_2_ + THP hydrate growing on a water droplet at *T*_ex_ = 282.1 K, Δ*T*_sub_ = 4.5 K, *P* = 4.95 MPa. The gas composition was H_2_:CO_2_ = 0.55:0.45 in mole fraction. The elapsed time after the hydrate nucleation is indicated below each image.

Observations of hydrate crystals on a water droplet are presented in Figs. 1, 2 and 3. Figure 1 shows sequential images of growing process of H_2_ + CO_2_ + THP hydrate at *T*_ex_ = 284.2 K, Δ*T*_sub_ = 1.3 K, *P* = 5.00 MPa. We determined *t* = 0 as the time when the nucleation of hydrate crystals was first identified visually. The hydrate nucleation was observed at the THP/water interface (Fig. 1a). As time proceeded, this nucleated hydrate crystal grew to form a polygonal plate along the THP/water interface (Fig. 1b). Subsequently, newly nucleated crystals were observed on the edge of the existing crystal plate (Fig. 1c). These hydrate crystals continued to grow and gradually covered the entire THP/water interface (Fig. 1d–f). After this time (*t* = 97 min), no noticeable further hydrate crystal growth was observed.

Although the time to hydrate formation process in this laboratory-scale system is quite long (97 min) for engineering practice, technical improvements could help shorten the time. For instance, installation of a process using micro-bubbles with a tubular reactor could enhance the hydrate formation rate 50 times^32^. In addition, hydrate nucleation rate could be improved with the 10^3^ of the size of a hydrate forming reactor.

Figure 2 shows sequential images of growing process of H_2_ + CO_2_ + THP hydrate at *T*_ex_ = 283.0 K, Δ*T*_sub_ = 2.5 K, *P* = 5.00 MPa. As recognized on the lower left of the images, hydrate crystals were nucleated and extended along the THP/water interface (Fig. 2a–c). At a subsequent time to the first nucleation, another nucleation was observed as recognized on the lower right of the image (Fig. 2b). These two lumps of hydrate crystals extended separately until they grew fully to collide, covering the entire droplet surface (Fig. 2c–e).

Figure 3 shows sequential images of growing process of H_2_ + CO_2_ + THP hydrate at *T*_ex_ = 282.1 K, Δ*T*_sub_ = 4.5 K, *P* = 4.95 MPa. At this raised Δ*T*_sub_, hydrate crystals grew and covered the THP/water interface rapidly after the first nucleation. As recognized in comparison of Figs. 1, 2 and 3, the time needed for the complete coverage of the droplet surface varies with the subcooling; when Δ*T*_sub_ is lowered, longer time is required. This difference of the elapsed time would be attributed to the difference of the hydrate nucleation rate and the crystal growth rate, both of which are affected by the driving force for crystal growth related to the magnitude of Δ*T*_sub_.

**Figure 4:**
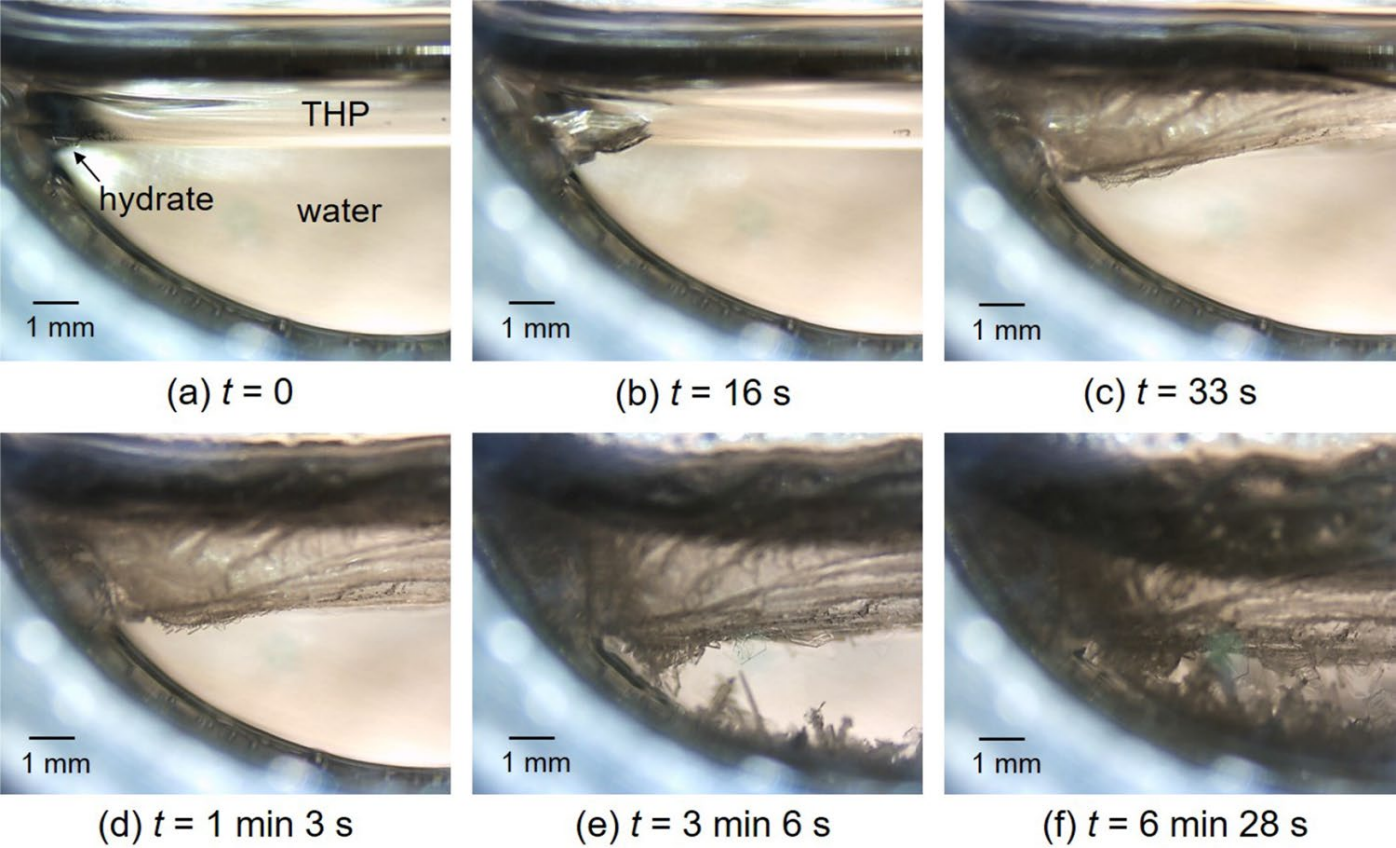
Sequential images of H_2_ + CO_2_ + THP hydrate growing in the liquid bulk at *T*_ex_ = 282.0 K, Δ*T*_sub_ = 5.1 K, *P* = 5.30 MPa. The gas composition was H_2_:CO_2_ = 0.55:0.45 in mole fraction. The elapsed time after the hydrate nucleation is indicated below each image.

Observations of hydrate crystals in a two-layer pool are presented in Fig. 4. Figure 4 shows sequential images of growing process of H_2_ + CO_2_ + THP hydrate at *T*_ex_ = 282.0 K, Δ*T*_sub_ = 5.1 K, *P* = 5.30 MPa. At *t* = 0, the hydrate nucleation was observed at the THP/water interface (Fig. 4a). This hydrate grain grew to form a pyramid (Fig. 4b) and gradually spread along the interface (Fig. 4c). After the complete coverage, the pyramidal crystal grew further into the bulk of water. Concurrently, minute hydrate grains sprouted up from the surface of the pyramidal crystal (Fig. 4d). These hydrate grains grew to form polygonal sheets, some of which detached from the surface (Fig. 4e). The lump of hydrate crystals covering the interface and the newly formed polygonal crystals grew larger (Fig. 4f).

This crystal growth behavior of hydrate formed in the liquid bulk exposed to H_2_ + CO_2_ is different from that of hydrate formed with CH_4_; in the CH_4_ + THP + water system, it is reported that hydrate is detached from the THP/water interface after the formation at the interface. Eventually, hydrate crystals which have been detached from the interface accumulate in the bulk of liquid water, without covering the THP/water interface^30^. In contrast, hydrate formed in this study promptly spread the entire THP/water interface before detaching (Fig. 4c). Therefore, it is inferred that the crystal growth rate of H_2_ + CO_2_ + THP hydrate at the interface is greater than that of CH_4_ + THP hydrate. One possible factor of this difference would be the gap of the interfacial free energies. The interfacial free energy in the CO_2_ + water system could be smaller than that in the CH_4_ + water system, resulting from the larger solubility of CO_2_ in water. Accordingly, the critical radius of H_2_ + CO_2_ + THP hydrate is considered smaller than that of CH_4_ + THP hydrate, both of the hydrates being structure II, which results in the increase of the nucleation rate and thereby the improvement of the formation rate.

### Crystal morphology

**Figure 5:**
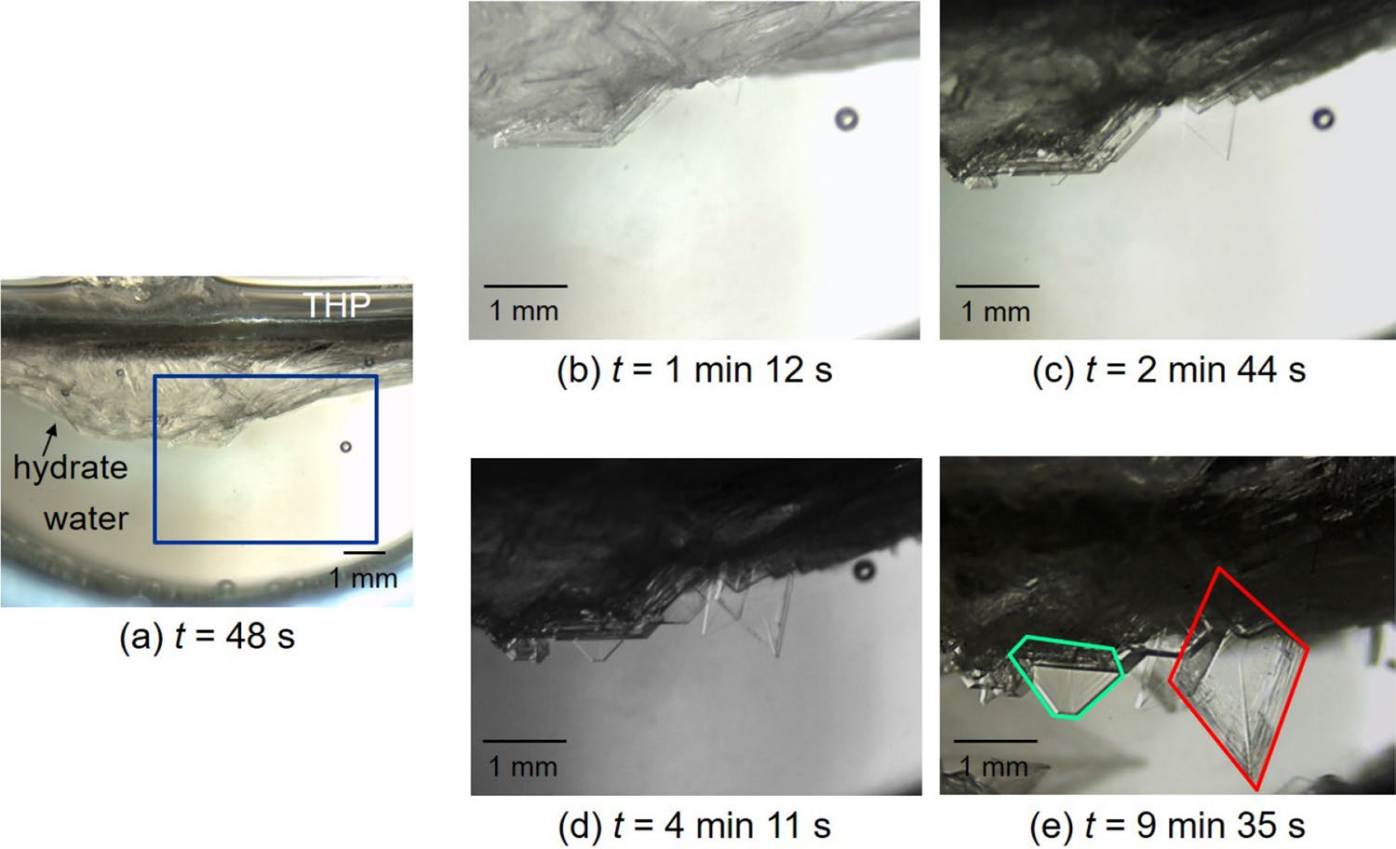
Sequential close-up images of H_2_ + CO_2_ + THP hydrate crystal plates growing in the bulk of liquid water at Δ*T*_sub_ = 4.8 K under *P* = 5.08 MPa. The gas composition was H_2_:CO_2_ = 0.65:0.35 in mole fraction. The elapsed time after the hydrate nucleation is indicated below each image.

We focus on hydrate single crystals formed at different levels of Δ*T*_sub_. Figure 5 shows temporal development of hydrate single crystals growing in the bulk of liquid water at Δ*T*_sub_ = 4.8 K under *P* = 5.08 MPa. Sequential images of enlarged part from Fig. 5a (enclosed by blue lines) are demonstrated in Fig. 5b–e. As recognized in Fig. 5b–e, the formation and growth of polygonal crystal plates were observed. Their high transparency would indicate that each plate was a hydrate single crystal. One crystal platelet grew to be hexagonal (emphasized by red lines) and another platelet formed into tetragonal (emphasized by green lines). These configurations may be attributed to the growing habit of hydrate crystals; it is reported that single crystals of cubic structure II hydrates grow to be octahedral or thin polygonal platelets^33–36^. Therefore, the observed hexagonal crystal plate exhibits a section parallel to the {111} plane of an octahedron. On the other hand, the observed tetragonal crystal plate is the “skeletal 60° plate” that Knight and Rider described^36^. According to their study, skeletal 60° plates of structure II hydrates appear when Δ*T*_sub_ are above 3 or 4 K in the THP + water system. This configuration is another typical form of structure II hydrates^34^. Although the mechanism of forming skeletal 60° plates is hard to explain in a crystallographic manner and is not revealed in the study by Knight and Rider^36^, visual observations of this study agree with the previous study. Thus, the observations would attest the fact that H_2_ + CO_2_ + THP hydrate is structure II hydate^37^.

**Figure 6:**
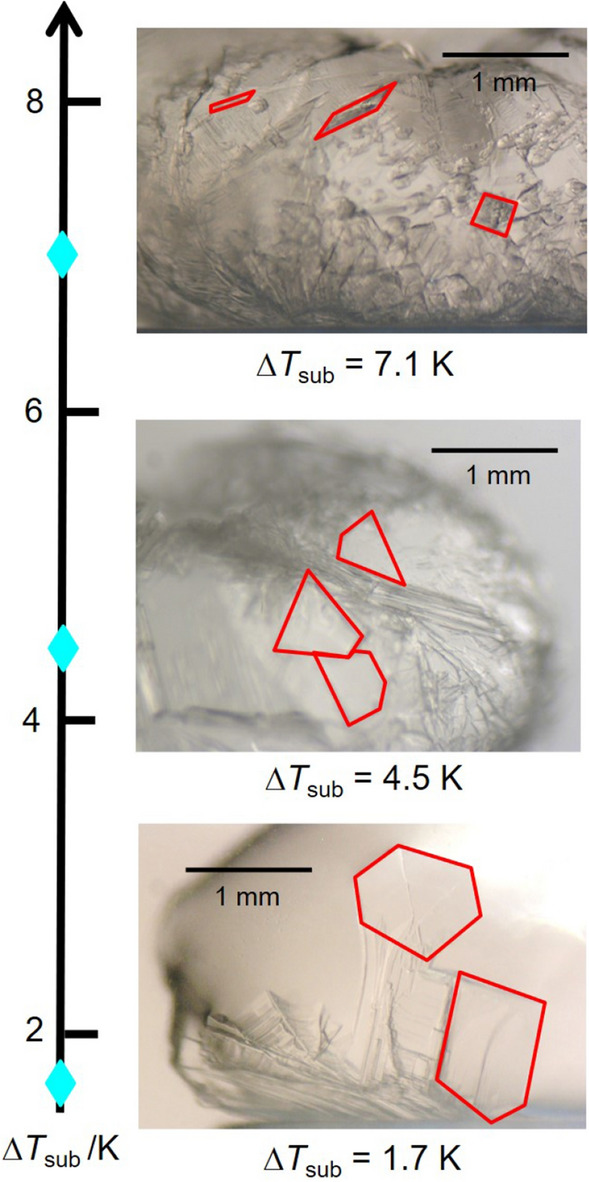
Arrangement of crystal morphology of H_2_ + CO_2_ + THP hydrate growing on a water droplet based on Δ*T*_sub_.

Figure 6 presents arrangement of morphology of hydrate crystal plates growing on a water droplet based on Δ*T*_sub_, where representative individual crystals are enclosed by red lines. At all Δ*T*_sub_, polygonal crystals were observed. The polygonal shape of plates may be a plane of an octahedron or demonstrate thin platelets. The difference depending on Δ*T*_sub_ was recognized in size of plates; as Δ*T*_sub_ rises, the side length of a single crystal plate decreases. Previous studies indicated that hydrate crystal morphology depends on the balance between the crystal nucleation rate and the crystal growth rate. When Δ*T*_sub_ increases, the nucleation rate rises and the number of crystals increases. Subsequently, growth of individual crystals is restricted since contact among crystals intensifies. Consequently, the size of crystals becomes small.

### Implication for industrial utilization

All of the experiments were conducted under the H_2_ + CO_2_ gas composition generally in consistent with that of systhesis gas produced from fuel sources, which is H_2_:CO_2_ = 0.6:0.4 in mole fraction. Therefore, the observations are to be applied to the hydrate-based CO_2_ separation process design. As described in the section **Crystal Growth Dynamics**, the time required for complete hydrate growth is shortened as Δ*T*_sub_ increases. This result concludes that the hydrate growth rate could be improved by the operation at a low temperature. On the other hand, resulted from the section **Crystal Morphology**, during the transportation process from the hydrate forming reactor to the hydrate dissociation vessel, smaller Δ*T*_sub_ is suitable. When the crystal size is large, the viscosity of hydrate slurry would decrease as derived from the rheological behavior, and the pressure loss becomes smaller^26^. Considering the dissociation process, larger Δ*T*_sub_ is appropriate. The surface area of hydrate crystals per unit volume increases as the size of polygonal crystals becomes smaller, which leads to enhanced heat and mass transfer. To determine the appropriate operating conditions, optimal value can be selected as a compromise.

From the obtained results suggesting that H_2_ + CO_2_ + THP hydrate is ready to form at the THP/water interface, it is concluded that hydrate formation methods with constant contact between THP and water would enhance the formation rate. Moreover, the observed successive water conversion to hydrate may represent sufficient crystal growth for the transportation process of hydrate slurry. For the continuous separation, water conversion ratio of approximately 10 ~ 20% is desirable to keep the fluidity of slurry.

The crystal morphology of H_2_ + CO_2_ + THP hydrate corresponds to those of other structure II hydrates observed in previous studies. Therefore, it is considered that the knowledge of structure II hydrate crystal growth that has been previously obtained can be applied to the hydrate-based separation process design.

Ricaurte et al.^11^ and Delroisse et al.^38^ reported that the combination of surfactant and organic compound enhances the water conversion into hydrate and thereby improves the CO_2_ separation efficiency in the batch system. Therefore, a combination of kinetic and thermodynamic promoters would provide a novel insight into hydrate-based continuous separation.

## Methods

**Figure 7:**
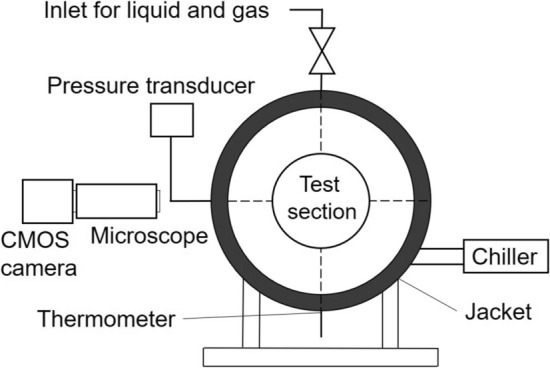
Schematic diagram of the experimental apparatus.

**Figure 8:**
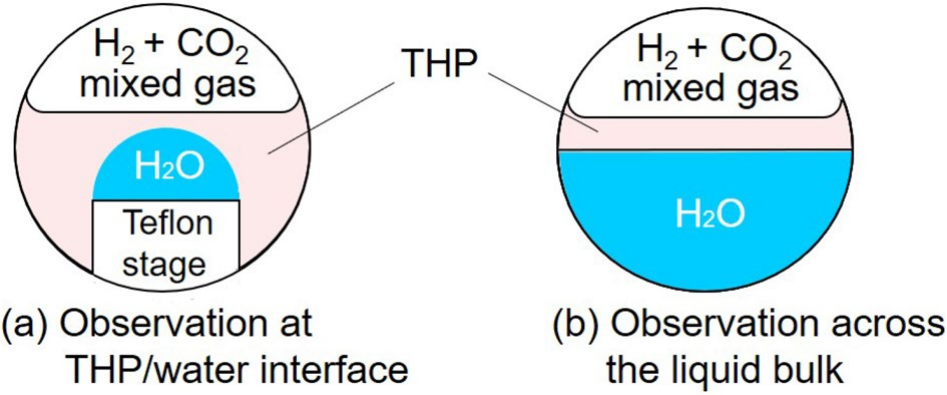
Schematic diagram of the test section.

The fluid samples used in the experiment were liquid tetrahydropyran (99 mass %, Aldrich Chemical Co.), H_2_ + CO_2_ mixed gas (CO_2_ certified purity of 39.7 vol %, Taiyo Nippon Sanso Co.), carbon dioxide gas (99.5 vol %, Taiyo Nippon Sanso Co.) and deionized, distilled water. Figure 7 shows the schematic diagram of the experimental apparatus. The test section is a stainless-steel cylindrical vessel with a pair of flange-type glass windows. The inner space of the test section is 25 mm in diameter and 20 mm in axial length. Figure 8a illustrates a frame format of the test section to observe hydrate at the liquid THP / liquid water interface. In this case, a cylindrical Teflon stage 6 mm in diameter was installed to hold a water droplet. Figure 8b describes a diagram of the test section to observe hydrate growing across the liquid bulk. The temperature inside the test section *T*_ex_ was controlled by circulating an ethylene glycol solution through a brass jacket covering the reactor, and measured by a Pt-resistance thermometer inserted from the undersurface of the reactor into the bulk of the liquid phase. The pressure inside the test section *P* was measured by a strain gauge pressure transducer. The estimated uncertainties of measurements were ± 0.2 K in temperature and ± 0.05 MPa in pressure. The formation and growth of hydrate crystals were observed and recorded using a CMOS camera (Aprolink Co.) and a microscope (Edmund Optics Co.).

The first process of the experimental procedure was the injection of liquid samples into the test section. For the observation of hydrate at the liquid THP/liquid water interface (see Fig. 8a), 4.0 cm^3^ of liquid THP was injected into the test section. Subsequently, a water droplet was placed on the Teflon stage, entirely immersed in the liquid THP phase. For the observation of hydrate growing across the liquid bulk (see Fig. 8b), 2.5 cm^3^ of liquid water and 0.5 cm^3^ of liquid THP were injected into the test section to form a two-layer pool. The air in the test section was then replaced with CO_2_ gas at about 1.0 MPa by repeating the process of pressurization and evacuation. H_2_ + CO_2_ mixed gas was charged in this vessel to approximately 5.5 MPa. Gases (primarily CO_2_) dissolved in liquids and *P* was settled to nearly 5.0 MPa in a steady state. After the completion of these procedures, *T* was lowered to approximately 270 K to form hydrate (and simultaniously ice). *T* was then increased stepwise by 0.1 K to experimentally determine the phase equilibrium temperature of the hydrate-forming system *T*_eq_ and to dissociate crystals. At each step, when no remarkable change of hydrate dissociation was observed within 10 min, *T* was increased. The temperature at which hydrate crystals were visually observed to dissociate rapidly was determined to be the equilibrium temperature *T*_eq_. After the dissociation of all hydrate crystals, *T* was set to the experimental temperature of crystal formation *T*_ex_ to form hydrate and to observe the hydrate formation and growth. After the observation, the H_2_ + CO_2_ gas composition was analyzed by a gas chromatograph. The equilibrium temperature measurement and the measurement of gas composition were conducted for each experimental run.

*T*_ex_ was set in the range from 279.5 to 284.9 K and *P* was in the range from 4.9 to 5.3 MPa. The reduction of system pressure resulted from the growth process of hydrate was less than 0.05 MPa, which is smaller than the uncertainty of pressure measurements.

## Supplementary Information

Supplementary Table.

## Data Availability

The datasets generated during and/or analysed during the current study are available from the corresponding author on reasonable request.
