# Supplementary material for: Crystal growth of clathrate hydrate formed with H2 + CO2 mixed gas and tetrahydropyran
Source: Sci Rep. 2021 May 31;11:11315. doi: 10.1038/s41598-021-90802-6 (PMC8167026; doi:10.1038/s41598-021-90802-6)
Supplement: Supplementary file 1 — Supplementary Table. [file 41598_2021_90802_MOESM1_ESM.pdf]

Title

"Crystal Growth of Clathrate Hydrate Formed with H_2 + CO_2 Mixed Gas and Tetrahydropyran"

Authors

Meku Maruyama†, Riku Matsuura†, Ryo Ohmura*†

*: corresponding author, E-mail: rohmura@mech.keio.ac.jp

†: Department of Mechanical Engineering, Keio University, 3-14-1 Hiyoshi, Kohoku-ku, Yokohama, Kanagawa 223-8522, Japan.

Table S1. Mutual Solubility[a] of organic compounds and water at T = 282.6 K, P = 0.1 MPa.

System	Water in organic x_w	Organic in water x_g	Mutual solubility $x_w + x_g$
THP + water	11.2×10^{-2}	2.3×10^{-2}	13.5×10^{-2}

[a] all the solubility values are expressed as mole fractions.
